# Global Trends in Green Space and Senior Mental Health Studies: Bibliometric Review

**DOI:** 10.3390/ijerph20021316

**Published:** 2023-01-11

**Authors:** Xialu Wu, Yu-Sheng Shen, Shenghui Cui

**Affiliations:** 1 Key Lab of Urban Environment and Health, Institute of Urban Environment, Chinese Academy of Sciences, Xiamen 361021, China; 2 Xiamen Key Lab of Urban Metabolism, Institute of Urban Environment, Chinese Academy of Sciences, Xiamen 361021, China; 3 University of Chinese Academy of Sciences, Beijing 100049, China

**Keywords:** landscape, environment, urban health, mental illness, psychological distress, well-being

## Abstract

The Sustainable Development Goals and the World Health Organization have prioritized senior mental health as an important goal. Senior mental health is a critical issue within the global public health sphere. Notably, green spaces are a useful alternative for improving senior mental health. Many studies have focused on green space and senior mental health, especially on their connection and relationship. However, this research topic lacks a comprehensive and systematic review. Owing to the lack of critical reviews, this study clarified the trend, progress, status, and focus of studies on green spaces and senior mental health using bibliometric analysis of literature within the Web of Science database. The literature analysis within this study specifically focused on the following, including the country/region contribution analysis, institution contribution analysis, keyword analysis, and highly productive journal analysis. Furthermore, this study systematically recorded the content of green space and senior mental health, identified the gap that exists, and provided future frontier directions or issues for research. These contribute toward comprehending the progress and content of this research topic and further provide a guide, reference, and inspiration for possible future research.

## 1. Introduction

By 2050, the world’s population of people aged 60 years and older will double (2.1 billion) [1]. Urban life stressors, such as crowding, noise pollution, fear of crime, and the economic burden caused by urbanization, threaten the mental health of senior residents and further increase the possibility of mental illness prevalence among senior residents [2,3]. In addition, the Sustainable Development Goals (SDGs) of the United Nations (UN) and the senior mental health promotion plan of the World Health Organization (WHO) regard senior mental health as an important goal and mission. Additionally, mental health and well-being are as important in older age as at any other time of life because mental and neurological disorders among older adults account for 6.6% of the total disability (DALYs) for this age group, and approximately 15% of adults aged 60 and over suffer from mental disorders [4]. Mental disorders in old age lead to impaired social functioning, decreased quality of life, and increased risk of health problems and comorbidities [5]. They have substantial social and economic impacts on families and societies, burdening health and social care services [6]. Thus, senior mental health is a critical issue in global public health.

Green spaces are open spaces covered by vegetation [7,8], which have ecological functions [9,10,11] and environmental functions (such as reducing air pollution and moderating heat) [12,13,14,15,16,17,18,19,20], as well as landscape functions accompanied by visual aesthetics [21]. Landscape and aesthetics can not only reduce people’s psychological stress and mental illness, but also improve happiness and satisfaction, promoting senior mental health [22]. Green spaces also provide extra space for residents to take part in physical activities, which encourages people to exercise more frequently and more intensely, which in turn promotes senior mental health [23,24,25,26,27,28,29]. Green spaces have positive effects on attention, mood, and physical activity; moreover, they play a unique role in promoting senior mental health [30,31]. Therefore, green spaces are a valuable alternative to improve seniors’ mental health [7].

Many studies have focused on green space and senior mental health, especially their linkage and relationship [23,24,29,32]. However, this research topic lacks a comprehensive and systematic review. Therefore, this study analyzed the literature on green space and senior mental health from the Web of Science (WoS) database and conducted a bibliometric analysis to assess the country/region contribution analysis, institution contribution analysis, keyword analysis, and highly productive journal analysis. Furthermore, this study systematically summarized the content of green space and senior mental health. It indicated its gaps and future frontier directions or issues for research. These can contribute to comprehending the progress and content of this research topic and provide a guide, reference, and inspiration for urban planning and healthy aging. Considering the special requests for green space from senior adults, the planning and policy of green space should consider the benefits and experiences of senior adults, such as the location, usability, and accessibility of green space. Moreover, policymakers can formulate a suitable greening policy for senior adults based on the results of this study.

## 2. Materials and Methods

The WoS is the most widely used database that provides high-quality journal publications. The Web of Science Core Collection (WoSCC) is one of WoS’s databases and includes SSCI, SCI-Expanded, ESCI, A&HCI, CPCI-S, CPCI-SSH, BKCI-S, and BKCI-SSH. Data were collected from the WoSCC database.

**Figure 1:**
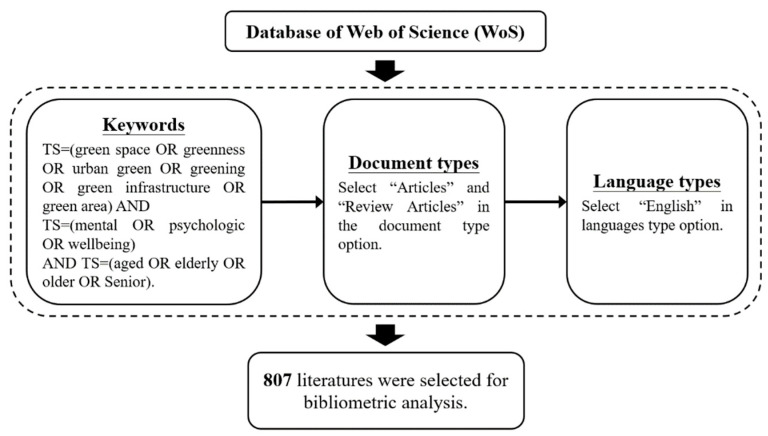
Flow chart of literature data collected for the study of urban green space and senior mental health. Note: TS means topic tag, which searches terms in title, abstract, author keywords, and keywords plus fields.

The data were collected in December 2022, and the collection process is illustrated in Figure 1. First, after testing numerous different keyword combinations, this study selected specific relevant literature by searching and combining the keywords related to “green space”, “mental health”, and “elderly”. Through the use of the Boolean operator tools of “OR” and “AND”, the search strategies were as follows: “TS = green space OR greenness OR urban green OR greening OR green infrastructure OR green area” AND “TS = mental OR psychologic OR wellbeing” AND “TS = aged OR elderly OR older OR Senior”. TS refers to a topic tag, which searches terms in the title, abstract, author keywords, and keywords plus fields. Second, the selected document types were peer-reviewed publications (i.e., “Article” and “Review Article”). Additionally, the language types in this study only included publications in English. Finally, this study obtained data from 807 studies via the aforementioned process.

Bibliometric analysis includes performance analysis and scientific mapping analysis, which can reveal research trends and clarify the current status of research. Performance analysis evaluates the influence of research from countries/regions, institutions, and authors. Scientific mapping analysis encompasses the dynamics and relationships observed within the research. Therefore, this study used bibliometric analysis based on WoSCC data to evaluate publication trends, country contributions, and institutional contributions. Moreover, the frequency of specific keywords and highly productive journals were analyzed using bibliometric analysis.

The general performance of publications was analyzed through Microsoft Excel. CiteSpace 6.1 R3 [33] was used to analyze countries/regions contribution and visualize the aforementioned studies. Keywords were analyzed and visualized through Bibliometrix, which is a R-tool for comprehensive science mapping analysis. The results of institution contribution analysis and high productive journals analysis were obtained from the WoSCC.

## 3. Results

### 3.1. General Performance of Publications

**Figure 2:**
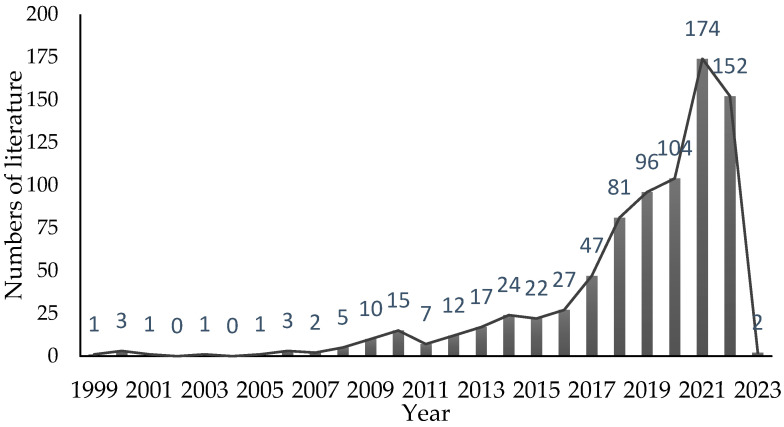
The trend of the number of annual publications for the study of green space and senior mental health.

A total of 807 studies on green space and senior mental health were collected via WoSCC. The selected literature was cited 21,208 times in total, with an average of 29.12 citations per literature, with roughly 25 studies recorded as being highly cited. Figure 2 shows the trend in the number of studies based on the concepts of “green space” and “senior mental health”. The number of publications has gradually increased since 2008, with a rapid growth trend beginning in 2016, and the largest increase between 2020 to 2021. This indicated that research on the topics of green space and senior mental health gradually became more popular from 2008, received high amounts of attention in 2016, and thereafter became a popular research topic in the fields of green space/greening landscapes. These increasing trends in research and publications were related to the SDGs from the UN and the advocacy for senior mental health from the WHO.

### 3.2. Countries/Regions Contribution Analysis

**Figure 3:**
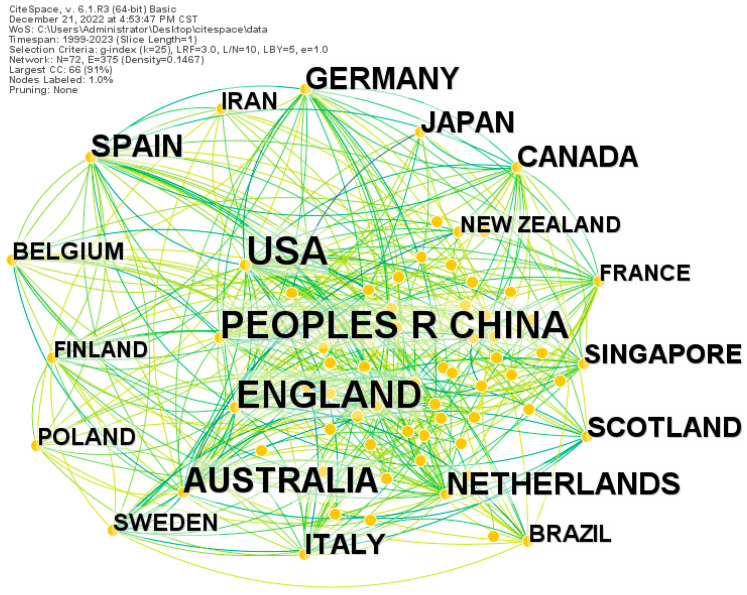
The geographical distribution and co-citation network for the study of green space and senior mental health. Note: The size of the label font in the figure represents the number of published documents, and the link between nodes represents the cooperative relationship between countries/regions.

This study assessed the status of green spaces and senior mental health in various countries/regions. Figure 3 illustrates the geographical distribution and co-citation network for the study of the topics of green space and senior mental health. The United States (US), China, and the United Kingdom (UK) are the top three countries with the highest number of research publications. Additionally, the US, the UK, Australia, the Netherlands, and Germany have exhibited strong causal links with other countries, indicating that researchers in these five countries have closely collaborated with researchers in other countries/regions on these research topics, further contributing to an increase in publications.

Table 1 below shows the top 20 countries/regions with the most publications surrounding the study of green space and senior mental health. The US, China, and the UK have more than 100 publications each, with the US, China, the UK, Australia, and the Netherlands as the top five countries with the highest number of publications. These results indicate that these countries have an abundance of high-intensity research papers and an area of focus in this research field. Additionally, according to the year of publication of the research papers, the US and France both started their study of green space and senior mental health before 2003, reflecting that these two countries focused on this research topic much earlier than other countries.

The geographical distribution of publications for the study of green space and senior mental health (Figure 3 and Table 1) shows 7 of the top 10 countries/regions are in North America and Europe, indicating that the research is conducted on these continents. Excluding the continents of North America and Europe, China (168) and Australia (81) are another two countries with a high number of publications on these research topics. Furthermore, the research quantity in China and Australia is higher than in other countries within their respective continents.

### 3.3. Institution Contribution Analysis

The contributions of institutions were vital for comprehending the research intensity and composition in this research field. Table 2 displays the performance of the top 10 institutions with the most publications on the study of green space and senior mental health. The achievements within this research field predominantly came from research institutes and universities. The top three achieving institutes with high quantitative research data were the Centro de Investigación Biomédica en Red/CIBERESP, Pompeu Fabra University, and the University of London.

### 3.4. Keywords Analysis

**Figure 4:**
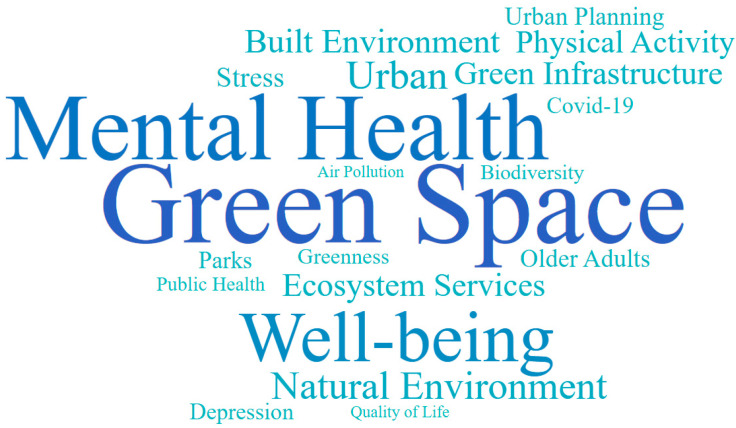
Word cloud of keywords for the study of green space and senior mental health. Note: The keyword size in the diagram represents the frequency of the keywords during searches.

This study revealed the focus and content of the study of green spaces and senior mental health using keyword analysis. Table 3 summarizes the top 20 keywords for the study of green spaces and senior mental health. All the keywords are “Green Space”, “Mental Health”, “Well-being”, “Urban”, “Natural Environment”, “Built Environment”, “Green Infrastructure”, “Ecosystem Services”, “Physical Activity”, “Stress”, “Older Adults”, “Urban planning”, “Parks”, “Depression”, “COVID-19”, “Greenness”, “Biodiversity”, “Public Health”, “Quality of Life”, and “Air Pollution”. Figure 4 shows a diagram of keywords based on the study of green spaces and senior mental health, as well as the frequency of the keywords.

### 3.5. High Productive Journals Analysis

Academic journals are vital for scientific research and play a role in the inheritance and dissemination of scientific achievements. This study found that 194 academic journals published papers related to the topics of green space and senior mental health. The top 10 journals for the study of green space and senior mental health are listed in Table 4. Table 4 lists the impact factors, SCImago journal rank, journal citation reports (JCR), and categories of the top 10 journals. Eight out of the top 10 countries were in JCR’s first quarter, revealing that most of the literature is of high quality and has scientific contributions.

## 4. Discussion

### 4.1. Research Trend, Development, and Hotspots

According to the bibliometric analysis results, research on the topics of green space and senior mental health gradually became more popular in 2008, received high amounts of attention in 2016, and thereafter became a popular research topic in the fields of green space/greening landscapes. According to the number of publications of the research papers, the US, China, the UK, the Netherlands, Australia, and Spain have an abundance of high-intensity research papers and an area of focus in this research field. Additionally, according to the year of publication of the research papers, the US and France focused on this topic much earlier than other countries. According to the results of the keyword analysis, stress and depression are the most common mental disorders, and the health benefits of green spaces are drawing immediate attention during the COVID-19 pandemic.

The mechanisms by which exposure to natural environments impacts our health have been suggested to include biopsychosocial pathways and are generally divided into three general functions: reducing harm (e.g., reducing exposure to air pollution, noise, and heat), restoring capacities (e.g., attention restoration and physiological stress recovery), and building capacities (e.g., encouraging physical activity and facilitating social cohesion) [34]. A previous study found that green spaces can provide restorative experiences and improve happiness and life satisfaction [35,36,37,38]. Individuals are found to be happier when living in urban areas with more green space in comparison to those who live in areas with less green space [39]. Maas et al. [40] indicated that green spaces were positively associated with separation from stress and mental fatigue. For instance, outdoor campus green spaces have been found to help students’ psychological [41], attention [42], and mental fatigue recovery [43].

Positive mental health among seniors is associated with parks with a natural focus and green spaces characterized by recreational and sporting activities containing adequate provisions of public green space in local neighborhoods [44]. Green spaces can provide people with space for physical activities, increasing the frequency and intensity of physical activities and further promoting senior mental health [32,45,46]. For example, Pretty et al. [47] evaluated the effects of 10 green exercise case studies (e.g., walking, cycling, horse-riding, fishing, canal-boating, and conservation activities) in 4 regions of the UK. This case study showed that green exercise could significantly improve self-esteem and mood disturbances, such as anger-hostility, confusion-bewilderment, depression-dejection, and tension-anxiety. However, it is difficult for older adults to engage in outdoor physical activity and have social connections with others during the pandemic because of the limited chances of exposure to public green spaces. Therefore, the mental health benefits of indoor green spaces have drawn immediate attention during the COVID-19 pandemic, and the relationship between indoor green spaces and senior mental health has become a research hotspot from 2020–2021. One study found that personal green spaces also reduced depression and anxiety levels of apartment residents during the COVID-19 pandemic [48]. Therefore, personal green spaces are as important as public green spaces for mental health benefits.

According to the results of the keyword analysis, urban is a highly mentioned keyword, which indicates that urban green space is the research focus. Previous studies have found that the higher the rate of greenery in a city, the less stress and fewer symptoms of depression reported among elderly residents [49]. In addition to the greenery of urban green spaces, the frequency of visiting urban green spaces also has a strong influence on seniors’ mental health. Previous studies have shown that more time spent in green space is associated with higher scores on senior mental health and vitality scales, and the more often a person visits urban open green spaces, the less often they will report stress-related illnesses [28,50]. Moreover, satisfaction with urban greenspace quality is an important predictor of well-being [51]. Urban greenspace quality was associated with a reduction in symptoms associated with psychological distress, including a dose-response relationship [52].

Stress and depression are also highly frequent, indicating that they are the most common mental disorders among senior adults and draw great public attention. Green spaces have been found to reduce the risk of mental illness among senior residents; the higher the levels of neighborhood green spaces, the lower the levels of symptomology associated with depression, anxiety, and stress [24]. Senior adults tend to go to nearby green spaces owing to their physical abilities, and it is difficult for them to reach green spaces over a long distance. Maas et al. [40] reported that the prevalence of symptoms of anxiety and depression was lower in living environments with more green space within a one km radius, especially for people of lower socioeconomic status. Braçe et al. [53] observed that using green spaces can help reduce the risk of experiencing symptoms associated with anxiety. Additionally, Nutsford et al. [29] reported that reduced distances to green spaces and increased size and quantity of green spaces within a community are associated with reducing the number of treatments for anxiety/mood disorders. Therefore, nearby green spaces are vital for seniors in obtaining mental health benefits.

### 4.2. Frontier of Green Space and Senior Mental Health

The frontier of green space and senior mental health includes: “the quantification of the elements from senior health-oriented green space planning”, “the equity of green space”, and “the long-term effects of green space on senior mental health”. Within “the quantification of the elements from senior health-oriented green space planning”, most research focused on the relationship between the area, coverage ratio, accessibility, and exposure of green space to elderly people’s mental health [29,36,44,54], and whether green space can improve or restore elderly people’s mental health [12,35,41,42,43]. There is extremely limited research on the quantitative criteria of the elements of senior health-oriented green space planning, such as quantity, fragmentation, dispersion, proportional coverage, area, and aggregation of green space. These quantitative criteria are critical for designing optimal senior health-oriented green spaces, and they need to be supported by scientific evidence. Therefore, based on the goal of senior health-oriented green space planning, it is necessary to explore the optimal elements of green spaces, including the quantitative criteria.

The concept of equity within “the equity of green space” is one of the goals of the SDGs, and it has also become a global concern in recent years. Some studies have explored the effects of green spaces on the mental health of senior residents of different sexes, ages, and socioeconomic conditions [55,56,57]. However, the research on the issues of equity within green spaces (e.g., fairness in the allocation of greening resources, the factors affecting the equity of green space, and the impact of green space equity on senior mental health) is still insufficient and needs to be explored in the future.

On the final topic of “the long-term effects of green space on senior mental health”, most previous studies on green space and senior mental health focused on short-term effects and current psychological status and reaction [39,44,51,54,58,59,60,61,62], and they overlooked the long-term effects of green space on senior mental health. Overall, a green space environment can continuously improve personal senior mental health [63]. Thus, the long-term effects of green spaces on personal senior mental health should be evaluated and quantified in the future.

### 4.3. Strengths and Limitations

This study has several strengths. First, this study explored the trends and status of the research on green spaces and senior mental health through bibliometric analysis. Moreover, this study systematically summarized the relationship between green space and senior mental health in previous studies. This study identified the gap and future frontier directions or issues for research on green spaces and senior mental health, which can provide a guide, reference, and inspiration for future research.

Finally, because English is the most used language for academic publications worldwide, this study only included publications in English. The limitation of this study is that it only analyzed English literature collected from the WoSCC database. Moreover, this study only considered peer-reviewed publications (i.e., “Article” and “Review Article”) on green space and senior mental health. In addition, the search terms might have excluded other relevant literature.

## 5. Conclusions

Research on green space and senior mental health is closely related to the SDGs and advocacy for senior mental health from the WHO, and it is a global research topic with high interest. Therefore, this study adopted bibliometric analysis to analyze the trends, progress, status, and hotspots of research on green spaces and senior mental health, including country/region contribution analysis, institution contribution analysis, keyword analysis, and high productive journal analysis.

Additionally, this study systematically summarized the content of green space and senior mental health. Furthermore, this study indicated future frontier directions or issues for research on green space and senior mental health, including the quantification of the elements of senior health-oriented green space planning, the equity of green space, and the long-term effects of green space on senior mental health. These can not only contribute to comprehending the progress and content of this research topic, but also provide a guide, reference, and inspiration for future research.

## Figures and Tables

**Table 1:** Top 20 countries/regions with the most publications surrounding the study of green space and senior mental health.

Rank	Country	Number of Publications	Starting Year	Rank	Country	Number of Publications	Starting Year
1	USA	181	1999	11	Italy	31	2010
2	China	168	2009	12	Singapore	26	2008
3	England	121	2006	13	Belgium	20	2014
4	Australia	81	2007	14	Sweden	20	2013
5	Netherlands	54	2010	15	Brazil	18	2012
6	Spain	52	2008	16	Poland	18	2019
7	Germany	46	2010	17	Iran	17	2018
8	Canada	41	2009	18	France	16	2003
9	Japan	40	2005	19	New Zealand	15	2009
10	Scotland	35	2013	20	Finland	15	2014

**Table 2:** Performance of the top 10 institutions with the most publications within the study of green space and senior mental health.

Rank	Institution	Country	Number of Publications				
			1900–2005	2006–2010	2011–2015	2016–2022	Total
1	Centro de Investigación Biomédica en Red/CIBERESP	Spain	0	1	6	52	59
2	Pompeu Fabra University	Spain	0	0	4	25	29
3	University of London	UK	0	2	6	19	27
4	ISGlobal	Spain	0	0	0	26	26
5	National University of Singapore	Singapore	0	2	5	15	22
6	Harvard University	USA	0	0	4	18	22
7	Utrecht University	Netherlands	0	1	0	21	22
8	University of California System	USA	0	0	2	19	21
9	University of Edinburgh	UK	0	0	3	18	21
10	Sun Yat Sen University	China	0	0	0	20	20

**Table 3:** Top 20 keywords for the study of green space and senior mental health.

Rank	Keywords	Frequency	Rank	Keywords	Frequency
1	Green Space	434	11	Older Adults	68
2	Mental Health	348	12	Urban Planning	68
3	Well-being	253	13	Parks	62
4	Urban	130	14	Depression	59
5	Natural Environment	123	15	COVID-19	57
6	Built Environment	102	16	Greenness	53
7	Green Infrastructure	98	17	Biodiversity	48
8	Ecosystem Services	96	18	Public Health	44
9	Physical Activity	95	19	Quality of Life	33
10	Stress	79	20	Air Pollution	31

**Table 4:** Top 10 journals for the study of green space and senior mental health.

Rank	Publications	Number of Publications	IF 2022	SJR 2022	JCR 2022	Categories
1	International Journal of Environmental Research and Public Health	113	4.614	Q1	Q1	Environment & Occupational Health (SSCI)
2	Urban Forestry & Urban Greening	47	5.766	Q1	Q1	Agricultural and Biological Sciences (SSCI); Environmental Science (SSCI); Forestry (SCIE);
3	Environmental Research	30	8.431	Q1	Q1	Environmental Science (SCIE); Public, Environment & Occupational Health (SCIE)
4	Health & Place	26	4.931	Q1	Q1	Public, Environment & Occupational Health (SSCI)
5	Landscape and Urban Planning	25	8.119	Q1	Q1	Ecology (SCIE); Environmental Studies (SSCI); Urban Studies (SSCI); Regional & Urban Planning (SSCI)
6	Environment International	23	13.352	Q1	Q1	Environmental Science (SCIE)
7	BMC Public Health	22	4.135	Q1	Q1	Public, Environment & Occupational Health (SCIE)
8	Sustainability	18	3.889	Q1	Q2	Social Sciences (SCIE); Environmental Science (SSCI)
9	PLoS One	16	3.752	Q1	Q2	Multidisciplinary Sciences (SCIE)
10	Frontiers in Psychology	15	6.461	Q1	Q1	Public, Environment & Occupational Health (SCIE&SSCI)

Note: IF: impact factors; SJR: SCImago journal rank; JCR: journal citation reports.

## Data Availability

Search keywords and collect literature from the database of web of science (accessed on 16 December 2022).
